# Peritoneal Inclusion Cysts in Female Children: Pathogenesis, Treatment, and Multimodality Imaging Review

**DOI:** 10.1155/2014/427427

**Published:** 2014-07-21

**Authors:** Rachelle Goldfisher, Divya Awal, John Amodio

**Affiliations:** Department of Radiology, SUNY Downstate Medical Center, 450 Clarkson Avenue, Brooklyn, NY 11203, USA

## Abstract

We report the multimodality imaging findings of peritoneal inclusion cysts in two adolescent females each with a prior history of abdominal surgery. The few reports of peritoneal inclusion cysts in the pediatric population have largely focused on the clinical and pathological features of this entity. We wish to emphasize the imaging findings of peritoneal inclusion cysts on multiple modalities, the advantage of MRI in confirming the diagnosis, and the need to keep considering this diagnosis in patients who present with a pelvic cystic mass, with a history of surgery, even if remote. Additionally, we review the pathology, pathophysiology, differential diagnosis, and treatment options of peritoneal inclusion cysts.

## 1. Introduction

Peritoneal inclusion cysts (PIC), also known as “peritoneal mesothelial cysts,” “peritoneal pseudocysts,” and “inflammatory cysts of the pelvic peritoneum,” have been described in females of reproductive age [1]. Reports of this entity occurring in adolescent females have largely focused on the clinical and pathological features of this entity [2, 3]. The literature describing the imaging findings of peritoneal inclusion cysts in the pediatric population on multiple modalities is scant. The majority of papers reports the incidence of PIC in the adult population and concentrates on imaging findings seen on sonography or CT [4, 5]. Familiarity with the radiological features of this benign lesion and its clinical associations is critical in making the diagnosis and may prevent invasive intervention. Furthermore, in cases where US and CT findings are equivocal, MRI should be considered for further characterization [4, 5]. MRI may delineate characteristic findings associated with PIC and may demonstrate the relationship of the cyst to the ovary more accurately.

Two cases of peritoneal inclusion cysts in patients with a history of abdominal surgery are presented. The first patient was born with genitourinary anomalies and underwent surgery three years prior to presentation. The second patient underwent surgery as an infant for necrotizing enterocolitis, and to our knowledge, this association has not been reported previously. The characteristic multimodality imaging findings associated with PIC are illustrated. We also review the pathogenesis, clinicopathological features, and differential diagnosis of PIC.

## 2. Case Reports

**Case 1 .**

**Figure 1:**
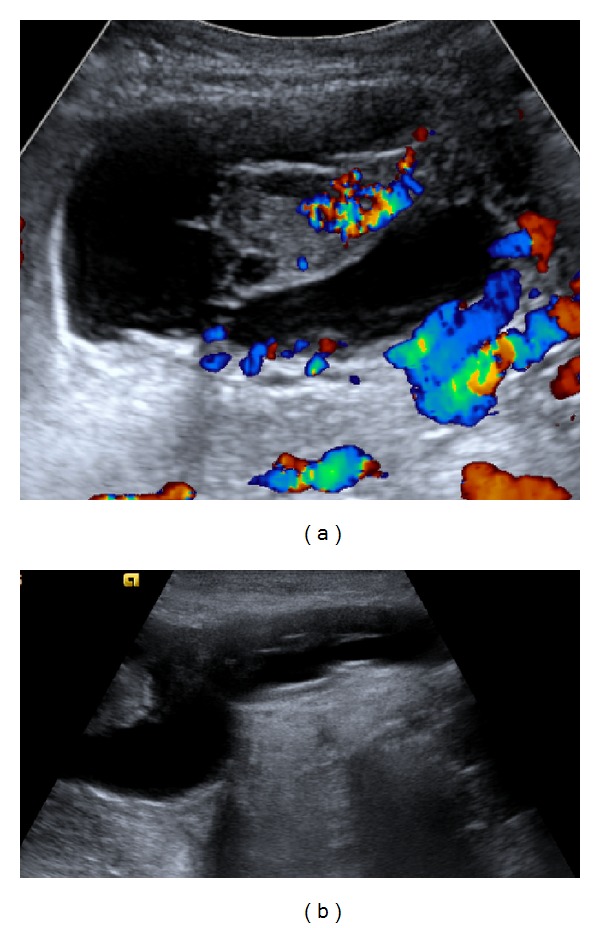
(a) Ultrasound demonstrating an avascular cystic collection in the right adnexal region, containing ovarian tissue that is connected to the peritoneum by a pedicle. (b) Ultrasound demonstrating extension of cystic mass into the right inguinal canal.

**Figure 2:**
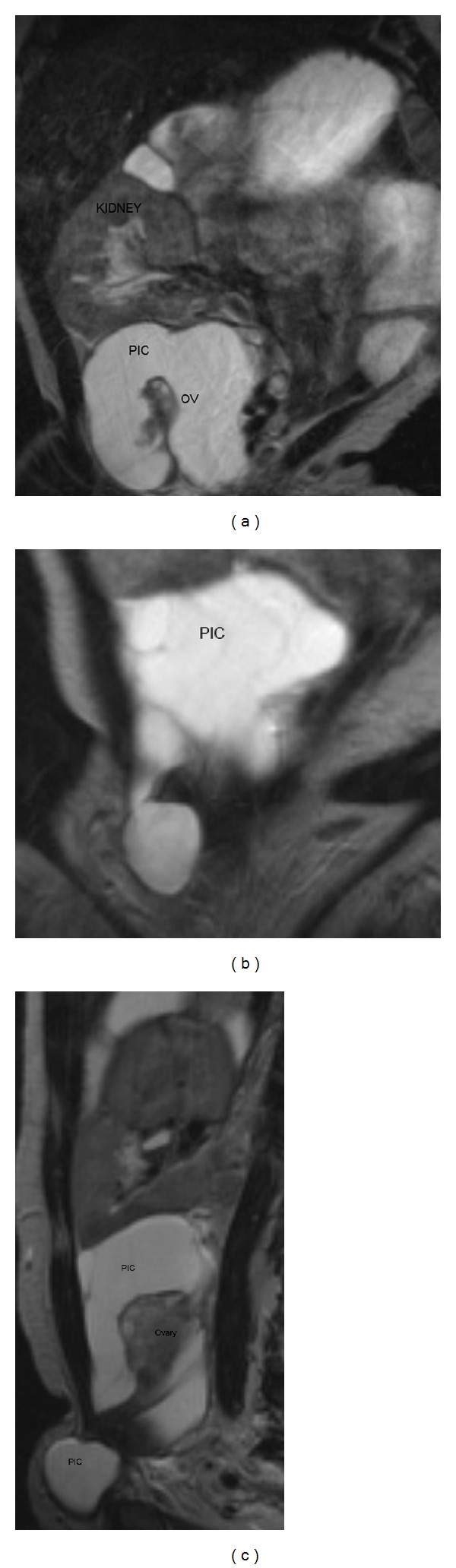
(a) Coronal T2 MR image demonstrating a cystic mass (PIC) containing ovarian tissue (OV) in the right adnexal region, extending into the inguinal canal. A transplanted right pelvic kidney is noted. (b) Coronal T2 MR image of peritoneal inclusion cyst (PIC) with extension to the right inguinal canal. Note “spider web” septations. (c) Sagittal T2 MR image of peritoneal inclusion cyst (PIC) with “spider web septations” containing ovarian tissue, with extension to the right inguinal canal.

A 16-year-old female presented with Mullerian renal cervical somite (MURCS) syndrome. The patient was born with ambiguous genetalia, multiple vertebral body segmentation anomalies, and unilateral renal agenesis. She presented three years after undergoing a right renal transplant with a painful right inguinal mass and increasing abdominal girth. Sonography demonstrated a large anechoic mass in the right lower quadrant extending into the right inguinal region (Figures 1(a) and 1(b)). The right ovary was visualized within the center of this collection attached via a vascular pedicle (Figure 1(a)). MR was performed for further characterization and delineation of adjacent anatomic structures. The MR demonstrated the relationship of this collection to the transplanted right pelvic kidney and the right ovary, which was enveloped within the cystic collection. The collection also demonstrated the characteristic “spider web” appearance of PIC (Figures 2(a), 2(b), and 2(c)). Therapeutic percutaneous drainage and biopsy were performed. Pathology revealed the collection to be compatible with a peritoneal inclusion cyst. Oral contraceptives were recommended to prevent recurrence; however the patient's mother refused. Two months later, the patient returned with a recurrence of a right peritoneal inclusion cyst.

**Case 2 .**

**Figure 3:**
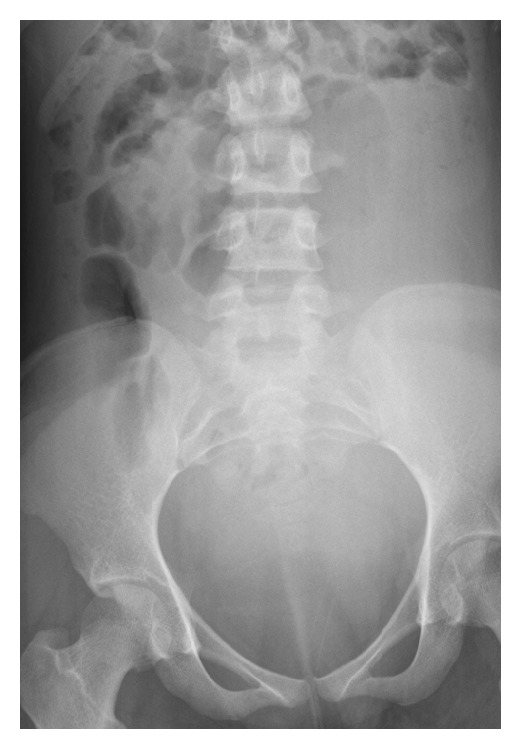
Abdominal radiograph demonstrating a large mass in the left lower quadrant, displacing bowel loops.

**Figure 4:**
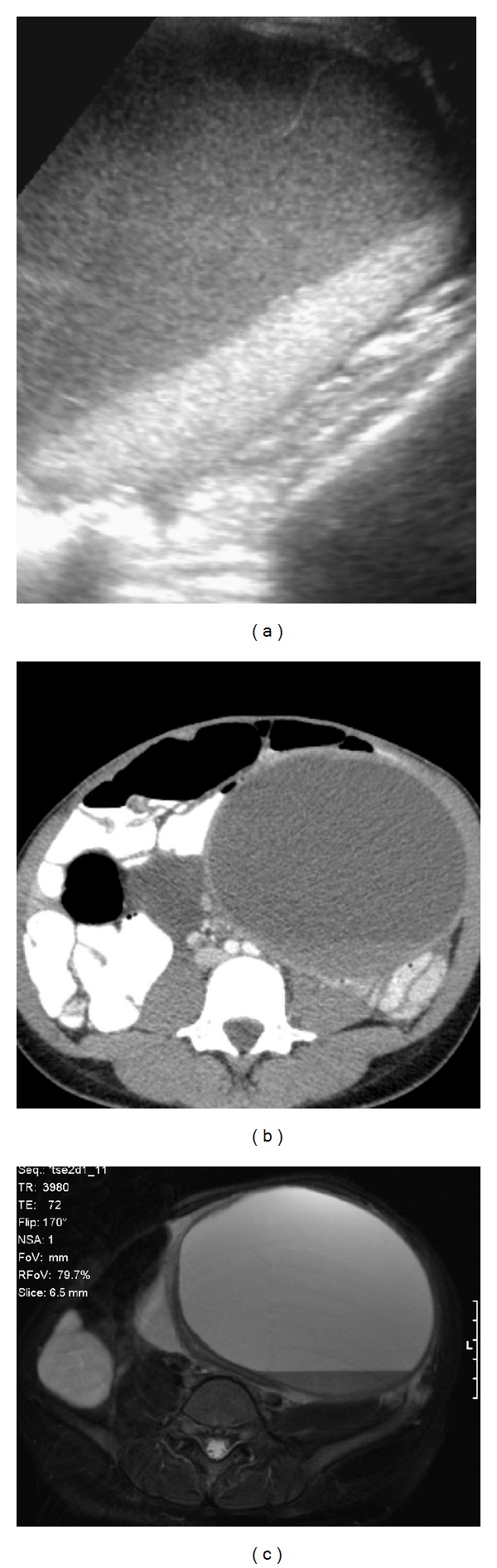
(a) Ultrasound demonstrating a complex cystic mass in the left adnexa with a blood fluid level and internal septations. (b) Axial CT demonstrating a large multiloculated left lower abdominal cystic “mass” with a blood fluid level. (c) Axial T2 MR image demonstrating a blood fluid level in a cystic “mass” in the left quadrant. There is also an additional component in the right lower quadrant.

**Figure 5:**
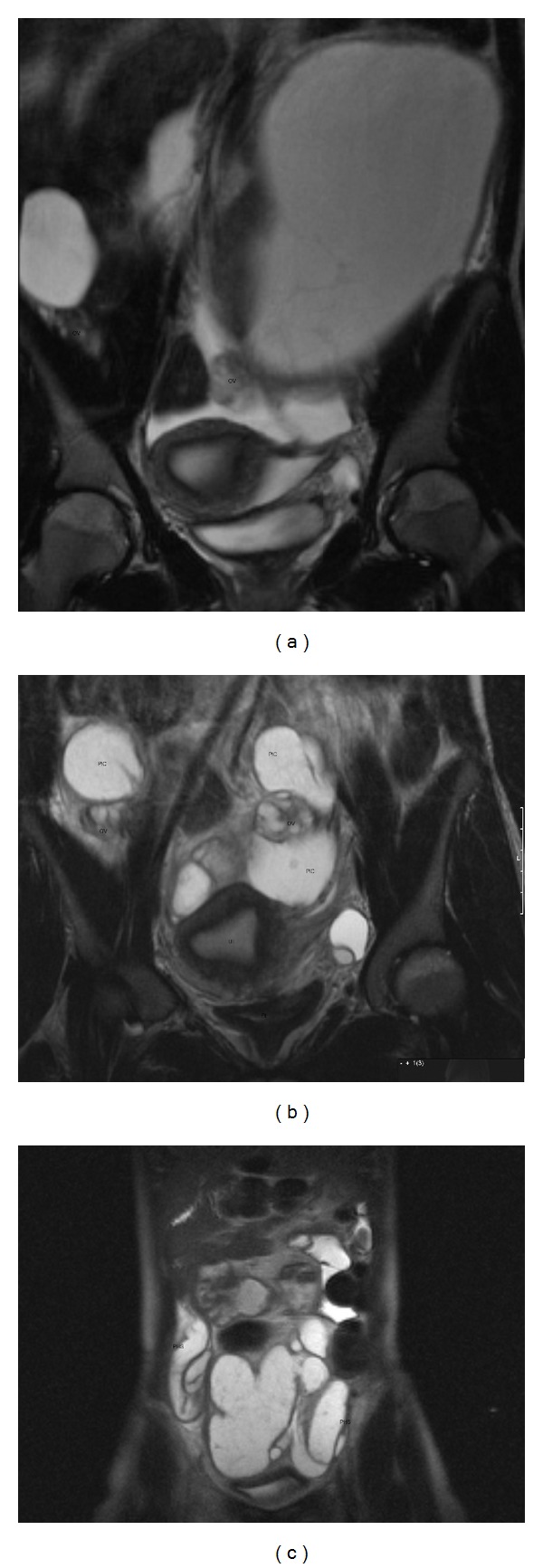
(a) Coronal T2 MR image demonstrating both ovaries (Ov) surrounded by cystic fluid collections. (b) Coronal T2 MR image shows both ovaries (Ov) surrounded by peritoneal inclusion cysts (PIC) after drainage. UT = uterus. Bl = bladder. (c) Coronal T2 MR image demonstrating a “pseudohydrosalpinx” (PHS) bilaterally. “Spider web” septations are seen within the peritoneal inclusion cyst that has decreased in size after drainage.

A 17-year-old female presented with left lower quadrant pain. She was a former 25-week premature infant and had undergone a partial colectomy during infancy for necrotizing enterocolitis. A radiograph was performed which demonstrated a paucity of bowel in the left lower quadrant and a large mass was suspected (Figure 3); an ultrasound was performed. An ultrasound demonstrated a large cystic mass with a dependent layer of echogenic material in the region of the left adnexa (Figure 4(a)). CT demonstrated a large cystic structure containing a fluid-fluid level with hemorrhagic material layering dependently (Figure 4(b)). The ovaries, however, were not visualized. MRI was performed for further evaluation, which demonstrated a large multiloculated cystic mass with a dependent blood-fluid level, separate from the ovaries. The patient underwent surgical drainage of the cystic collection. Pathology at that time revealed the cystic mass to be a hemorrhagic peritoneal inclusion cyst. Subsequently, the “mass” returned, at which an additional MRI was performed. The ovaries were identified and were surrounded by smaller cystic fluid collections consistent with smaller peritoneal inclusion cysts. Interestingly, some of the collections were tubular, resembling hydrosalpinx (Figures 5(a), 5(b), and 5(c)).

## 3. Discussion

During the reproductive years, fluid produced by the ovaries is normally absorbed by the peritoneum. When the integrity of the peritoneum is disrupted as a result of surgery, trauma inflammation, or endometriosis the peritoneum has decreased ability to absorb fluid. In addition, postsurgical adhesions can trap ovarian fluid that is no longer being absorbed by a disrupted peritoneum, producing a complex cystic pelvic mass. Functional, active ovaries and adhesions are thus essential for the development of peritoneal inclusion cysts, compatible with the presentation of this lesion upon and after the onset of puberty.

The most common presenting symptom in patients with a peritoneal inclusion cyst is lower abdominal or pelvic pain. On physical exam, often there are no palpable abdominal or pelvic masses [2]. Common causes for the development of peritoneal inclusion cysts include postsurgical adhesions, pelvic inflammatory disease, endometriosis, and trauma. Endometriosis is less common in the pediatric age group. The time between the most recent surgery and the detection of PIC has been reported to range from 6 months to 20 years. In one series of adolescent females with PIC, appendectomy was the most common prior surgery [2].  Case 2 in this report illustrates the need to keep this diagnosis in mind even with a remote history of surgery.

Peritoneal inclusion cysts are simple or complex cystic adnexal collections consisting of a normal ovary entrapped in multiple fluid filled adhesions. Grossly, the cysts are often multiple with some forming confluent masses. Individual locules may contain serous fluid, gelatinous fluid, or hemorrhagic material [6].

Pathologically, peritoneal inclusion cysts are pseudocysts and are typically lined by hyperplastic mesothelial cells proliferating within inflamed fibrous granulation tissue walls [4]. Often, the ovary is connected to the peritoneum by a pedicle [1]. The trapped ovary, producing physiologic fluid, is mostly responsible for the fluid within these cysts; the surrounding inflammation may cause an exudate, resulting in persistence and growth of PIC in patients on hormonal treatment [1].

Most cases of PIC express focal negative immunoreactivity for CEA. This needs to be considered when entertaining a diagnosis of PIC versus an ovarian malignancy. Rarely, however, PIC may exhibit positive CEA immunostaining [2].

As previously mentioned, peritoneal inclusion cysts may not be palpable and may only be detected via imaging [7]. Thus, the imaging features of these cysts are important for detection and diagnosis.

Ultrasound is the most frequently used modality in the initial workup of pelvic pain, as it does not use a radiation source, it is relatively inexpensive, and it is easily accessible. In addition, sonography may be useful in lesions warranting image-guided aspiration. The most typical sonographic finding of a peritoneal inclusion cyst is a normal ovary surrounded by anechoic fluid containing multiple septations [8]. The fluid can be echogenic secondary to hemorrhagic or proteinaceous material. When adhesions surround the ovary and fluid accumulates forming a cystic mass, the trapped ovary often has the appearance of what has been described as a “spider in a web” [3, 9].

On CT, peritoneal inclusion cysts depict a cystic mass with regular or irregular borders, containing material with the attenuation properties of fluid and/or hemorrhage. MR images demonstrate cystic lesions with low T1 signal and high T2 signal consistent with serous fluid. In cases of hemorrhagic material, cysts may demonstrate high T1 signal. The high contrast resolution of soft tissues on MR makes it useful in detecting these pseudocysts. The shape of peritoneal inclusion cysts may be irregular, secondary to mass effect by adjacent organs, as these pseudocysts lack true walls. Contrast-enhanced T1 images typically demonstrate little or no contrast enhancement. Again, the “spider in a web” appearance of the lesion on MRI is typical of PIC. MRI may also be helpful in defining the extraovarian nature of the fluid collection(s), as the ovary may not be clearly defined on sonographic examination, as in Case 2.

The main differential diagnosis for PIC in the pediatric population includes macrocystic lymphatic malformation, hydrosalpinx/pyosalpinx, paraovarian cysts, and ovarian epithelial malignancy. Macrocystic lymphatic malformations appear as multilocular cystic masses that are anechoic or contain internal septations and echogenic debris. There may be enhancement of the septations within the lymphatic malformation, not characteristically seen in PIC. Hydrosalpinx may mimic a peritoneal inclusion cyst, as adhesions can entrap fluid in an oblong loculation adjacent to the uterus, as illustrated in (Figure 5(c)). Occasionally, peritoneal inclusion cysts can contain an echogenic fluid collection with a tubular configuration simulating the appearance of pyosalpinx. A distinguishing feature of PIC versus hydro/pyosalpinx is that the ovary is typically encompassed within the fluid, with multiple septations from the ovary. However, clinical history may be needed to aid in the correct diagnosis. Paraovarian cysts may mimic PIC when fluid is noted adjacent to the cyst in the adnexa. True paraovarian cysts appear as single or multiple cystic masses separate from the ovary; they are characteristically found in the broad ligament. Identification of a normal ipsilateral ovary separate from the cyst, in a patient with no prior surgery, is helpful in diagnosing a paraovarian cyst. Additionally, paraovarian cysts usually do not have thickened walls or septa [2]. Although PICs may have a complex appearance, ovarian epithelial malignancy would be expected to have enhancement after the administration of contrast on MRI, not typical for a PIC. Additionally, the ovary, if identified, would not have a normal appearance in cases of epithelial malignancy.

Endometrioma may have a similar appearance to PIC on sonographic examination and also should be considered in the differential diagnosis. However, an endometrioma usually has hemorrhagic components, not typically seen in PIC. Again, MRI may be helpful in distinguishing an endometrioma from PIC.

Both conservative and surgical management approaches have been described in patients with a peritoneal inclusion cyst. The use of oral contraceptives is thought to decrease the amount of fluid produced by the ovary. As reported by Hoffer et al. [6], women with severe endometriosis and those with inactive ovaries or those taking oral contraceptives produce less peritoneal fluid. Indications for adhesiotomy would include infertility or persistence and recurrence of the lesion in patients on oral contraceptives. Surgical options include open resection, laparoscopic drainage, and percutaneous drainage. The risk of recurrence after extensive surgical resection has been reported to be 30–50% [1].

In summary, we emphasize the importance of entertaining the diagnosis of a peritoneal inclusion cyst in pediatric patients with a history of surgery and in whom a simple or complex cystic mass is demonstrated on imaging. Although sonography plays a major role in the diagnosis, MRI is a useful adjunct in demonstrating ovarian tissue entrapped within the cyst. Familiarity with the characteristic imaging findings of PIC and knowledge of its clinical associations is useful to establish this diagnosis and guide appropriate treatment and may avoid aggressive intervention.
